# Supplementary material for: Spontaneous rotation can stabilise ordered chiral active fluids
Source: Nat Commun. 2019 Feb 22;10:920. doi: 10.1038/s41467-019-08914-7 (PMC6385212; doi:10.1038/s41467-019-08914-7)
Supplement: Supplementary file 1 — Supplementary Information [file 41467_2019_8914_MOESM1_ESM.pdf]

Spontaneous rotation can stabilise ordered chiral active fluids: Supplementary material

Ananyo Maitra[1,*] and Martin Lenz[1,2,†]

[1] LPTMS, CNRS, Univ. Paris-Sud, Université Paris-Saclay, 91405 Orsay, France
[2] MultiScale Material Science for Energy and Environment, UMI 3466, CNRS-MIT, 77 Massachusetts Avenue, Cambridge, Massachusetts 02139, USA

Supplementary Note 1. Introduction

In this supplement, we will present the equations of motion for apolar and polar chiral systems in detail, look at the dynamics of defects in an apolar system and finally study the effects of concentration fluctuations. In section Supplementary Note 2, we will consider the dynamics of apolar chiral fluids in a two-dimensional momentum conserved film, in a two-dimensional film exchanging momentum with a three-dimensional fluid and finally, a two-dimensional film in contact with a substrate which acts as a momentum sink. In section Supplementary Note 3 we perform the equivalent calculations for a polar system. In section Supplementary Note 4 we study the dynamics of isolated disclinations in an apolar chiral fluid and show that intrnsic particle rotation can suppress defect separation. Finally, in section Supplementary Note 6 we study the coupled dynamics of concentration and polar or apolar order parameters within a pertubation scheme that we discuss and show that in most cases the number fluctuations of this active system scale the same way as passive ones.

Supplementary Note 2. Two-dimensional apolar systems

In this section we discuss the dynamics of an active apolar chiral system. We first study apolar chiral systems in two-dimensional momentum conserved free-standing films Supplementary Note 2 a, and as in the main text, use this to introduce the calculation. We then discuss the differences in cases where the momentum is dissipated in a three-dimensional fluid Supplementary Note 2 b or to a substrate Supplementary Note 2 c.

a. two-dimensional momentum conserved systems – free-standing film

In this subsection we construct the equations of motion of chiral apolar particles in a free-standing two-dimensional film and demonstrate that intrinsic particle rotation can stabilise a rotating orientationally ordered phase. The free-energy functional governing the equilibrium dynamics of apolar systems irrespective of the form of momentum dissipation (i.e whether the momentum is conserved in a two-dimensional film or whether it is dissipated in three-dimensional fluid or into a substrate) is given by a Landau-de Gennes functional which we write in a single Frank constant approximation:

$$\mathcal{H} = \int d^2\mathbf{r}[(\alpha/2)\mathsf{Q} : \mathsf{Q} + (\beta/4)[\mathsf{Q} : \mathsf{Q}]^2 + (K/2)(\nabla\mathsf{Q})^2] \tag{1}$$

The dynamical equation for the orientation tensor is

$$D_t\mathsf{Q} = \lambda\mathsf{A} - \lambda_c\boldsymbol{\epsilon}\cdot\mathsf{A} - \Gamma_Q\mathsf{H} - 2\Omega\boldsymbol{\epsilon}\cdot\mathsf{Q}. \tag{2}$$

where D_t denotes the corotational derivative, A is the symmetric part of the velocity gradient tensor: $A_{ij} = \partial_i v_j + \partial_j v_i$, and $\mathsf{H} = \delta\mathcal{H}/\delta\mathsf{Q}$. The first term on the R.H.S describes the tendency of the apolar particles to orient along the velocity gradient and the third describes its relaxational dynamics in absence of flow and activity. There are two explicit chiral terms in (2): $\boldsymbol{\epsilon}\cdot\mathsf{A}$ is a chiral coupling between orientation and flow that is present even in chiral passive fluids and $\Omega\boldsymbol{\epsilon}\cdot\mathsf{Q}$, arising from microscopic rotation of the chiral particles, which is forbidden in passive systems, but is allowed here. This term is responsible for the global rotation of the ordering direction at a constant rate. As discussed in the main text, this is ultimately responsible for the stabilisation of the ordered phase.

The evolution of the velocity field is governed by the Stokes equation for the slow flows that we are interested in. The deviations from the ordered state generate both active and passive forces in the system. The passive force densities are completely determined by Onsager symmetry and are proportional to $\nabla\cdot\mathsf{H}$ and $\nabla\cdot(\boldsymbol{\epsilon}\cdot\mathsf{H})$ because of

microscopic time-reversal symmetry. They are subdominant to the active force densities that we will discuss next for fluctuations about an ordered phase and thus, we ignore them here. The active force densities must be a divergence of a symmetric tensor because of angular momentum conservation. The symmetric tensor must be a function of $\mathbf{Q}$, and to zeroth order in gradients, there are two of them in a chiral system: one simply proportional to $\mathbf{Q}$, and another proportional to $\boldsymbol{\epsilon} \cdot \mathbf{Q}$. The former is the standard active stress allowed in both chiral and achiral active system [1], while the latter is allowed only in chiral systems since the presence of $\boldsymbol{\epsilon}$ implies that it breaks mirror symmetry. The forces arising from orientation fluctuations are balanced by the ones arising from pressure gradients and viscous dissipation. Thus, the force balance equation finally reads

$$-\eta\nabla^2\mathbf{v} = -\nabla\Pi + \zeta\nabla\cdot(\mathbf{Q}) - \zeta_c\nabla\cdot(\boldsymbol{\epsilon}\cdot\mathbf{Q}), \tag{3}$$

where η is the viscosity and Π is the pressure that enforces the incompressibility constraint $\nabla\cdot\mathbf{v} = 0$.

We now investigate an ordered phase with a constant $S = S_0 = 1$. In a state without any spatial gradients of $\mathbf{Q}$ we see from (2) that the angle field rotates at its intrinsic rotation rate $\partial_t\theta = \Omega$. Perfect alignment of particles also implies the perfect synchronisation of their phases. Thus, the alignment direction of the perfectly aligned phase rotates in space with an angular speed Ω.

We now seek to study the stability of this phase to angular fluctuations. For this, we write $\theta(\mathbf{r}t) = \Omega t + \delta\theta(\mathbf{r}, t)$. Solving for the velocity in terms of the angle field, we find the spatially Fourier-transformed equation for the angular fluctuations

$$\partial_t\delta\theta_q = -\frac{1}{2\eta}[\zeta\cos 2(\phi-\Omega t) + \zeta_c\sin 2(\phi-\Omega t)][1+\lambda\cos 2(\phi-\Omega t) - \lambda_c\sin 2(\phi-\Omega t)]\delta\theta_q + \mathcal{O}(q^2) \tag{4}$$

where $\mathbf{q} = q(\cos\phi\hat{x} + \sin\phi\hat{y})$ is the wavevector. For $\Omega \approx 0$, the relaxation rate is always negative for some ϕ, as pointed out in the main text.

The general solution of this differential equation is obtained from the Floquet theory in the forn

$$\delta\theta_q(t) = e^{\mu t}\Phi(t)\delta\theta_q(0)\,; \qquad \Phi\left(t + \frac{2\pi}{\Omega}\right) = \Phi(t) \tag{5}$$

where

$$\mu 2\pi/\Omega = \int_0^{2\pi/\Omega} -\frac{1}{2\eta}[\zeta\cos 2(\phi-\Omega t) + \zeta_c\sin 2(\phi-\Omega t)][1+\lambda\cos 2(\phi-\Omega t) - \lambda_c\sin 2(\phi-\Omega t)]dt \tag{6}$$

which immediately gives us the Lyapunov exponent

$$\mu = -\frac{\zeta\lambda - \zeta_c\lambda_c}{4\eta}, \tag{7}$$

and Φ is a periodic function in time whose maximum value scales as $e^{\nu/\eta\Omega}$ where ν is a function of ζ, ζ_c, λ and λ_c. When $\mu < 0$, the maximum growth of $\delta\theta_q$ is bounded by the maximum of Φ and $\delta\theta_q$ goes to 0 at large times. This maximum growth is small if $\Omega \gg \zeta/\eta$ and ζ_c/η. Therefore, in this limit $\delta\theta_q$ is asymptotically stable and its maximal growth even at short times is small. This implies that in this case the rotating ordered phase will not be destroyed by fluctuations as we pointed out in the main text.

b. Two-dimensional monolayer in the interface between three-dimensional fluids

In this subsection we study the dynamics of a chiral apolar monolayer at the interface between two three-dimensional bulk fluids, which we take to have the same viscosity. We further ignore all vertical fluctuations of the interfacial layer. The field $\mathbf{Q}$ describes the two-dimensional apolar order parameter as earlier. The three-dimensional force-balance equation is

$$-\eta\boldsymbol{\nabla}^2\mathbf{V} = -\boldsymbol{\nabla}\mathcal{P} + \zeta\nabla\cdot(\mathbf{Q})\delta(z) - \zeta_c\nabla\cdot(\boldsymbol{\epsilon}\cdot\mathbf{Q})\delta(z), \tag{8}$$

where $\mathbf{V} = \mathbf{V}_\perp + V_z\hat{z}$ denotes the three-dimensional fluid velocity with $\hat{z}$ being the direction normal to the interface, $\boldsymbol{\nabla}$ denotes the three-dimensional, while ∇ denotes the two-dimensional gradient and $\mathcal{P}$ is the three-dimensional pressure

enforcing the incompressibility condition $\mathbf{\nabla} \cdot \mathbf{V} = 0$. Define the Fourier transform of the velocity field as

$$\mathbf{V}_\perp = \int \frac{dq^3}{(2\pi)^3} \mathbf{V}_{\perp_q} e^{-i\mathbf{q}\cdot\mathbf{R}} \tag{9}$$

where $\mathbf{R}$ is the three-dimensional position vector. The dynamical equation for the apolar order parameter is given by (2) with the identification

$$\mathbf{v}_q = \mathbf{V}_{\perp_q}|_{z=0} = \int_{-\infty}^{\infty} \frac{dq_z}{2\pi} \mathbf{V}_{\perp_q} \tag{10}$$

Solving for $\mathbf{V}_q$ using (8) and calculating $\mathbf{v}_q$ from (10) we obtain the equation for angular and concentration fluctuations.

$$\partial_t \delta\theta_q = -\frac{|q|}{8\eta}[\zeta \cos 2(\phi - \Omega t) + \zeta_c \sin 2(\phi - \Omega t)][1 + \lambda \cos 2(\phi - \Omega t) - \lambda_c \sin 2(\phi - \Omega t)]\delta\theta_q + \mathcal{O}(q^2) \tag{11}$$

For large Ω, we can again use the procedure in Supplementary Note 2 a to calculate the Lyapunov exponent which is

$$\mu = -|q| \frac{\zeta\lambda - \zeta_c\lambda_c}{16\eta} \tag{12}$$

as presented in the main text.

c. Two-dimensional monolayer on a substrate

In this subsection, we show that the presence of a momentum sink in the form of a substrate renders the relaxation of the orientation fluctuations diffusive while still allowing for an actively stabilised QLRO phase. While the form of the apolar order parameter equations remain the same in this case, the form of the force balance equation changes in two distinct ways: 1. the presence of a wavevector independent damping and 2. a new active force depending on angular fluctuations. The force balance equation, to the lowest order in gradients, is

$$\Gamma \mathbf{v} = -\nabla \Pi + \zeta \nabla \cdot \mathbf{Q} - \zeta_c \nabla \cdot \boldsymbol{\epsilon} \cdot \mathbf{Q} - 2\zeta_2 \mathbf{Q} \cdot (\nabla \cdot \mathbf{Q}), \tag{13}$$

which features three active terms, as opposed to only two in earlier cases due to the lack of momentum conservation. Again, eliminating velocity, we obtain

$$\partial_t \delta\theta = -q^2 \left[\frac{1}{2\Gamma} \{\zeta_2 + \zeta \cos 2(\phi - \Omega t) + \zeta_c \sin 2(\phi - \Omega t)\}\{1 + \lambda \cos 2(\phi - \Omega t) - \lambda_c \sin 2(\phi - \Omega t)\} + \Gamma_Q K \right] \delta\theta. \tag{14}$$

As earlier, in the fast-rotating case, the Lyapunov exponent can be calculated to be $-q^2[(2\zeta_2 + \lambda\zeta - \lambda_c\zeta_c)/4\Gamma + \Gamma_Q K]$. Note that a relaxation rate $\propto q^2$ implies that in two dimensions the angular fluctuations diverge logarithmically at large scales upon the addition of noise i.e. the phase only has QLRO.

Supplementary Note 3. Two-dimensional polar systems

In this section, we consider each of the three scenarios considered in section Supplementary Note 2 for polar instead of apolar ordering. We will show that while the stability of the polar ordered state in a two-dimensional momentum conserved film Supplementary Note 3 a and a film in contact with a three-dimensional fluid Supplementary Note 3 b parallel those found for their apolar counterparts in sections Supplementary Note 2 a and Supplementary Note 2 b, that of a polar phase in contact with a substrate Supplementary Note 3 c is distinct from the corresponding apolar one discussed in section Supplementary Note 2 c.

a. two-dimensional momentum conserved systems – free-standing film

The dynamics of a polar system in the absence of activity and flow is governed by the free-energy

$$\mathcal{F} = \int d^2\mathbf{r}(\alpha_p/2)p^2 + (\beta_p/4)p^4 + (K_p/2)(\nabla\mathbf{p})^2, \tag{15}$$

where $\mathbf{p}$ is the polarisation vector that measures the degree of local polarisation in the system, irrespective of the form of the momentum dissipation mechanism. For the polar phase to be realised, $\alpha_p < 0$.

The dynamical equation for the polarisation field is given by

$$D_t\mathbf{p} + \lambda_1\mathbf{p}\cdot\nabla\mathbf{p} + \lambda_2(\boldsymbol{\epsilon}\cdot\mathbf{p})\cdot\nabla\mathbf{p} = \lambda\mathbf{p}\cdot\mathsf{A} - \lambda_c\mathbf{p}\cdot(\boldsymbol{\epsilon}\cdot\mathsf{A}) - \Gamma_p\mathbf{h} - \Omega\boldsymbol{\epsilon}\cdot\mathbf{p}, \tag{16}$$

where λ_1 describes polar active self-advection and λ_2 describes a chiral self-advection and $\mathbf{h} = \delta\mathcal{F}/\delta\mathbf{p}$. Note that both λ_1 and λ_2 as well as the other possible self-advective terms we have not explicitly retained (of the form $\mathbf{p}(\nabla\cdot\mathbf{p})$ etc.; see [2, 3] etc.) since they do not affect the linearised theory, lead to corrections to the Lyapunov exponent at $\mathcal{O}(q)$ whereas, as in the apolar fluid, the term corresponding to λ result in the leading $\mathcal{O}(q^0)$ behaviour of the Lyapunov exponent.

The force-balance equation is given by

$$-\eta\nabla^2\mathbf{v} = -\nabla\Pi + \zeta\nabla\cdot(\mathbf{pp}) - \zeta_c\nabla\cdot[\boldsymbol{\epsilon}\cdot(\mathbf{pp})]. \tag{17}$$

Note that this is similar to (3) if one identifies Q with $\mathbf{pp}$ (the isotropic part of the latter tensor can be absorbed into a redefined pressure and does not lead to any flow in an incompressible system). The lowest order terms that break $\mathbf{p}\to-\mathbf{p}$ symmetry are $\nabla^2\mathbf{p}$ and $\nabla^2(\boldsymbol{\epsilon}\cdot\mathbf{p})$. Since they are subdominant to the apolar terms, we will not consider them further,

Deep in the polar phase, the fluctuations of the polarisation magnitude decay fast to its steady state value p_0 which we take to be 1. The equation of motion for the angular fluctuations is given by

$$\partial_t\delta\theta = -\frac{1}{2\eta}[\zeta\cos 2(\phi-\Omega t)+\zeta_c\sin 2(\phi-\Omega t)][1+\lambda\cos 2(\phi-\Omega t)-\lambda_c\sin 2(\phi-\Omega t)]-[\lambda_1\cos(\phi-\Omega t)-\lambda_2\sin(\phi-\Omega t)]iq-\Gamma_pK_pq^2. \tag{18}$$

In the fast-rotating case, this leads to the same Lyapunov exponent as in Supplementary Note 2 a as discussed in the main text.

b. Two-dimensional monolayer in the interface between three-dimensional fluids

We now consider a polar version of the system described in Supplementary Note 2 b and use a similar procedure to find the evolution equation for the angular fluctuations:

$$\partial_t\delta\theta = -\frac{|q|}{8\eta}[\zeta\cos 2(\phi-\Omega t)+\zeta_c\sin 2(\phi-\Omega t)][1+\lambda\cos 2(\phi-\Omega t)-\lambda_c\sin 2(\phi-\Omega t)]-[\lambda_1\cos(\phi-\Omega t)-\lambda_2\sin(\phi-\Omega t)]iq-\Gamma_pK_pq^2 \tag{19}$$

In the fast-rotating case, this leads to the same Lyapunov exponent as in Supplementary Note 2 b as discussed in the main text.

c. Two-dimensional monolayer on a substrate

Unlike the previous two cases, the polar chiral fluid on a substrate, composed of swimmers rotating in circles, in phase, near a substrate, is quite distinct from its apolar counterpart discussed in Supplementary Note 2 c. The lack of momentum conservation in this case ultimately leads to the Lyapunov exponent for the angular fluctuations scaling as $\mathcal{O}(q^0)$ and not as $\mathcal{O}(q^2)$. This leads to a remarkably stable long-range-ordered state. A similar behaviour in an achiral polar system has been discussed in [4].

The difference from the apolar system in this case is due to new couplings between the polarisation vector and the velocity that are allowed due to the lack of momentum conservation. The lack of Galilean invariance implies that

polarisation can respond to not only the gradient of velocity but also to the velocity itself

$$\partial_t \mathbf{p} = \Lambda \mathbf{v} - \Lambda_c \boldsymbol{\epsilon} \cdot \mathbf{v} - \Gamma_p \mathbf{h} - \Omega \boldsymbol{\epsilon} \cdot \mathbf{p}, \tag{20}$$

where the passive couplings Λ and Λ_c denote the response of the polarisation vector to velocity, and not its gradient, in systems without Galilean invariance. The former is present in achiral systems as well, while the latter is only present in chiral ones. The advective, self-advective and flow-alignment terms that appeared in Supplementary Note 3 a are not explicitly shown here since they appear at higher order in gradients and are therefore subdominant at large scales.

To lowest order in gradients, the force balance equation is

$$\Gamma \mathbf{v} = \upsilon \mathbf{p} - \upsilon_c \boldsymbol{\epsilon} \cdot \mathbf{p} - \nabla \Pi, \tag{21}$$

where the first term on the R.H.S denotes active motility of polar particle and the second, a chiral motility. Π enforces incompressibility, as before. We have neglected passive forces arising from polarisation fluctuations, as well as active forces at higher order in gradients, which are subdominant at large scales compared to the two motilities.

Unlike in Supplementary Note 3 a and Supplementary Note 3 b, the perfectly ordered phase in this case has a spontaneous, non-vanishing velocity field. This implies that the ordered phase sets in when $\bar{\alpha}_p = \alpha_p + (\Lambda_c \upsilon_c - \Lambda \upsilon)/\Gamma$ turns negative and not when $\alpha_p < 0$. The magnitude of the order parameter in this case is $|\mathbf{p}| = p_0 = \sqrt{|\bar{\alpha}_p|/\beta}$. The ordering direction rotates at an angular speed $\Omega_p = \Omega + (\Lambda_c \upsilon + \Lambda \upsilon_c)/\Gamma$.

Deep in the ordered phase, when the magnitude of polarisation is essentially constant, the angular fluctuations have the dynamical equation

$$\partial_t \delta\theta = \left[-\frac{\Lambda \upsilon}{\Gamma} \sin^2(\phi - \Omega_p t) + \frac{\Lambda \upsilon_c - \Lambda_c \upsilon}{2\Gamma} \sin 2(\phi - \Omega_p t) + \frac{\Lambda_c \upsilon_c}{\Gamma} \cos^2(\phi - \Omega_p t) \right] \delta\theta. \tag{22}$$

In the fast-rotating case, the Lyapunov exponent to order q^0 is

$$\mu = -\frac{\Lambda \upsilon - \Lambda_c \upsilon_c}{2\Gamma} \tag{23}$$

as discussed in the main text. Note that unlike the apolar system Supplementary Note 2 c, the relaxation rate of fluctuations in this case does not vanish in the limit of infinite system sizes which implies the existence of a long-range ordered state.

Supplementary Note 4. Chiral Defects

In this section, we consider the dynamics of defects in a chiral active nematic and show that intrinsic particle rotation can suppress defect separation which can destroy achiral active nematics at high activities.

It is known that $+1/2$ defects in achiral active nematics self-propel due to active forces [5, 6]. This propulsion is due to the backflow generated by a defect configuration. We now examine how the self-propulsion of defects is affected by chirality in an apolar fluid.

The active forces in a chiral apolar fluid are

$$\mathbf{f} = [\zeta \nabla \cdot \mathbf{Q} - \zeta_c \nabla \cdot (\boldsymbol{\epsilon} \cdot \mathbf{Q})]. \tag{24}$$

They can be written in terms of the angle field θ using

$$\nabla \cdot \mathbf{Q} = S \begin{pmatrix} -\sin 2\theta & \cos 2\theta \\ \cos 2\theta & \sin 2\theta \end{pmatrix} \begin{pmatrix} \partial_x \theta \\ \partial_y \theta \end{pmatrix}, \tag{25}$$

$$\nabla \cdot (\boldsymbol{\epsilon} \cdot \mathbf{Q}) = S \begin{pmatrix} \cos 2\theta & \sin 2\theta \\ \sin 2\theta & -\cos 2\theta \end{pmatrix} \begin{pmatrix} \partial_x \theta \\ \partial_y \theta \end{pmatrix} \tag{26}$$

when S is a constant. In Equilibrium, defects are singular solutions of the Euler-Lagrange equations that minimise the Landau-de Gennes free-energy. In the single Frank-constant approximation, this implies that they are solutions

of the Laplace equation. A defect at the origin can be parametrised as

$$\theta = \theta_0 + \frac{n}{2}\psi \tag{27}$$

in polar coordinates, where ψ is the polar angle and n is an integer. The order parameter magnitude S is a constant outside the core region, which we assume to be small. In nematics, defects with charge $\pm 1/2$ i.e. $n = \pm 1$ are energetically most favourable.

In a chiral spontaneously rotating system, out of equilibrium, the angle field configuration corresponding to a charge $\pm 1/2$ isolated disclination is

$$\theta = \Omega t \pm \frac{1}{2}\psi. \tag{28}$$

We will now calculate the backflow due to this. Far away from the defect core, when $S = S_0 = 1$,

$$\nabla \cdot \mathbf{Q} = \frac{n}{2r}[\cos\{(1-n)\psi - 2\Omega t\}\hat{x} - \sin\{(1-n)\psi - 2\Omega t\}\hat{y}] \tag{29}$$

$$\nabla \cdot (\boldsymbol{\epsilon} \cdot \mathbf{Q}) = -\frac{n}{2r}[\sin\{(1-n)\psi - 2\Omega t\}\hat{x} + \cos\{(1-n)\psi - 2\Omega t\}\hat{y}] \tag{30}$$

where r is the distance from the defect core (origin). Therefore, the active force for a $+1/2$ defect is

$$\mathbf{f}_{+1/2}(r,\psi) = \frac{1}{2r}[\zeta\{\cos(2\Omega t)\hat{x} + \sin(2\Omega t)\hat{y}\} - \zeta_c\{\sin(2\Omega t)\hat{x} - \cos(2\Omega t)\hat{y}\}] \tag{31}$$

Now, following Mishra et al., we can can calculate the velocity field due to this defect using the Oseen tensor $\mathcal{G}_{ij}(\mathbf{r}-\mathbf{r}')$ appropriate for two-dimensional momentum conserved flows (other systems will have the same qualitative behaviour), in a circular domain of radius R:

$$v_i(\mathbf{r}) = \int_{0<r<R} d\mathbf{r}' \mathcal{G}_{ij}(\mathbf{r}-\mathbf{r}')\mathbf{f}_{+1/2_j}. \tag{32}$$

This implies that

$$\mathbf{v}_{+1/2}(0,\psi) = \frac{R}{4\eta}[\zeta\{\cos(2\Omega t)\hat{x} + \sin(2\Omega t)\hat{y}\} - \zeta_c\{\sin(2\Omega t)\hat{x} - \cos(2\Omega t)\hat{y}\}] \tag{33}$$

It has been shown that when the flow is much faster than the orientational dynamics of the angle field, the core of the $+1/2$ defect is simply advected by $\mathbf{v}_{+1/2}(0,\psi)$ [5–9]. This implies that the core of a $+1/2$ defect in a rotating chiral system rotates in a circle of radius $R\sqrt{\zeta^2+\zeta_c^2}/(8\eta\Omega)$. Therefore, its velocity, averaged over a period is 0 implying that it doesn't move ballistically, as mentioned in the main text.

If the system is chiral but non-rotating, i.e. $\Omega = 0$, the velocity is given by

$$\mathbf{v}_{+1/2} = \frac{R}{4\eta}[\zeta\hat{x} + \zeta_c\hat{y}] \tag{34}$$

This implies that a defect will move at an angle $\tan^{-1}(\zeta_c/\zeta)$ with respect to the direction of its "nose". Of course, if, in addition, $\zeta_c = 0$, the system becomes completely achiral and this angle goes to 0, as expected for achiral systems. Experimentally, measuring the direction of motion of the defect with respect to its orientation can be useful in determining the ratio of the chiral and achiral force densities as we pointed out in the main text.

Supplementary Note 5. Deterministic dynamics of a $\pm 1/2$ defect configuration

In this section we will examine the deterministic dynamics of a $\pm 1/2$ defect pair. The separation between the $\pm 1/2$ defect pairs is denoted by $\mathbf{r} = (x\hat{x} + y\hat{y}) = r(\cos\varphi, \sin\varphi)$, and the polarisation of the $+1/2$ defect is denoted by $\mathbf{e} = \nabla \cdot \mathbf{Q}(+) = |\mathbf{e}|(\cos\xi, \sin\xi)$, where $\nabla \cdot \mathbf{Q}(+)$ represents the value of $\nabla \cdot \mathbf{Q}$ at the location of the $+1/2$ defect. Using the standard force between a $\pm 1/2$ defect pair, $-(\pi K/2r)\hat{r}$, we write down the deterministic translational dynamics

when the magnitude of $\mathbf{e}$ is assumed to be fixed:

$$\dot{\mathbf{r}} = v\hat{\mathbf{e}} - v_c \boldsymbol{\epsilon}\cdot\hat{\mathbf{e}} - \mu\frac{\pi K}{2r}\hat{r} \tag{35}$$

where $v = R\zeta/4\eta$ and $v_c = R\zeta_c/4\eta$ and $\mu = s\Gamma_Q$ is a defect mobility, where s is a non-universal (depending on the details of the defect core) numerical constant. The calculation of the torque on the $+1/2$ defect orientation is much more complicated. In principle, it has to be calculated from dynamics of the apolar order parameter $\mathbf{Q}$. Such a calculation was performed in an active achiral system by [14]. In a chiral system similar considerations lead to a orientational dynamics

$$\dot{\xi} = 2\Omega - \frac{s\pi v}{16r}\sin(\xi-\varphi) - \frac{s\pi v_c}{16r}\cos(\xi-\varphi) \tag{36}$$

We now rewrite (35) in terms of the coordinates r and φ:

$$\dot{r} = v\cos(\xi-\varphi) - v_c\sin(\xi-\varphi) - \mu\frac{\pi K}{2r} \tag{37}$$

$$r\dot{\varphi} = v\sin(\xi-\varphi) + v_c\cos(\xi-\varphi) \tag{38}$$

Defining $\delta = \xi - \varphi$ as the angle that the $+1/2$ defect polarisation makes with the line joining the $+1/2$ and the $-1/2$ defects, we obtain the coupled dynamics

$$\dot{\delta} = 2\Omega - \left(1+\frac{s\pi}{16}\right)\frac{v}{r}\sin\delta - \left(1+\frac{s\pi}{16}\right)\frac{v_c}{r}\cos\delta \tag{39}$$

$$\dot{r} = v\cos\delta - v_c\sin\delta - \mu\frac{\pi K}{2r} \tag{40}$$

We first discuss these coupled equations in the absence of chirality. This case was fully analysed by [14]. In the absence of chirality, $\delta = 0, \pi$. That is, for $v < 0$, $\delta = \pi$ is the stable fixed point of the angular dynamics while for $v > 0$, $\delta = 0$ is the stable point. Since the distances are measured from the $-1/2$ to the $+1/2$ defect, $\delta = \pi$ implies that the defect orientation aligns *with* the direction of the attractive force, as pointed out in [14] while $\delta = 0$ implies anti-alignment. From the r dynamics we then find that activity pushes the defects apart and (through the torque) ensures that the defects move apart in a straight line. Ref [14] demonstrated that this picture of active defect separation is complicated by the inclusion of noise. Noise randomises the direction of motion of the $+1/2$ defect, and in the process can arrest this activity-driven unbinding of defects. However, achiral nematics are destroyed by defect unbinding both at high activities *and* at low noise.

Considering now a chiral defect in the *absence* of rotation i.e. when $\Omega = 0$, for the case when $v > 0$ and $v_c > 0$, we immediately see that the dynamics is similar to the achiral case – the active backflow pushes the $+1/2$ defect away in a straight line from the $-1/2$ defect. However, the orientation of the defect makes a *fixed* angle $\delta = \tan^{-1}(-v_c/v)$ to the line joining the $\pm1/2$ defects. In Fig. Supplementary Figure 1 we show that the distance between the $\pm1/2$ defect increases with time.

For $\Omega \neq 0$, this picture is distinctly modified. In this case, at large distances from the $-1/2$ defect (i.e., $r \to \infty$), the $+1/2$ defect rotates with a radius $\sqrt{v_c^2+v^2}/2\Omega$ as we discussed in the last section. The deterministic dynamics of the defect recombination is however more complicated (to say nothing of the stochastic dynamics). First, it is clear that when the separation $r \ll \pi\mu K/2v$ and $\pi\mu K/2v_c$, a $+1/2$ defect recombines with its $-1/2$ partner irrespective of the direction of defect polarisation i.e., at this scale, the angular dynamics of the defect ceases to matter. The active orienting torques significantly affect the dynamics only at separations $r \ll v/2\Omega$ and $v_c/2\Omega$ (while writing these relations, we have taken $1 + s\pi/16 \approx 1$ for simplicity. In our numerical calculation of defect trajectories, we set $s = 1$). At larger separations, the δ can be taken to be simply $2\Omega t$. This implies that when $\Omega \gg v^2/\pi\mu K$ and $v_c^2/\pi\mu K$, the active orienting torques are only relevant at scales at which the $+1/2$ defect recombines irrespective of its orientation. Therefore, in this case we can safely ignore the active orienting torques everywhere and take $\delta = 2\Omega t$. This is the case we discussed in the main text. In this case, since the active velocities in (40) average to 0, the $+1/2$ defect recombines deterministically with the $-1/2$ defect irrespective of the initial separation. We present some trajectories of such recombining defects for various parameters in Fig. Supplementary Figure 2. We note that all the parameters other than Ω in Fig. Supplementary Figure 1 and Fig. Supplementary Figure 2 are the same. This demonstrates

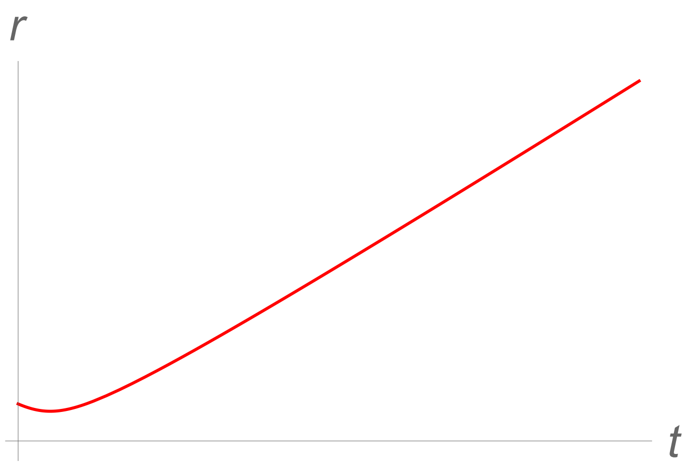

Supplementary Figure 1: The distance between a chiral $\pm 1/2$ defect pair with time when $\Omega = 0$.

that turning on chiral rotation leads to deterministic recombination of defects in a parameter-regime where without rotation, the $+1/2$ and $-1/2$ defects would have separated at least at low noise. The situation is more complicated when $\Omega \ll v^2/\pi\mu K$ or $v_c^2/\pi\mu K$. In this case (assuming that the ordered state is not linearly unstable even at this Ω) the angular torques are relevant at scales at which the attractive interaction does not dominate the ballistic active motion of the defect. This can lead to $\dot{r}$ and $\dot{\delta}$ vanishing at a finite δ_0 and at a distance r_0 from the $-1/2$ defect which much larger than $\pi\mu K/2v$ and $\pi\mu K/2v_c$. This implies that in this case the $+1/2$ defect rotates (recall that δ is the angle between the defect polarisation and azimuthal angle) at a constant angular speed around the $-1/2$ defect. Note that this r_0 is distinct from the radius of the circular trajectory of the $+1/2$ defect in the absence of the $-1/2$ defect. We have checked, by expanding (39) and (40) about r_0 and δ_0 that such bound circular orbits can remain stable for a range of parameters. Even when the circular orbit is not stable, the $+1/2$ defect can essay quasi-periodic almost circular orbits around the $-1/2$ defect and remain within a finite distance of it. We will discuss the detailed dynamics of such bound defect pairs, as well as the stochastic dynamics of pair recombination elsewhere. However, even in the case where the $\pm 1/2$ defects form a bound state, they remain within a finite distance of each other at all times and the global orientational order at large scales is not destroyed by defect-unbinding. This validates our assertion that activity-induced unbinding of defects may be suppressed in the spontaneously rotating phase and unlike active nematics in which defect-unbinding inevitably leads to a low noise isotropic phase, ordering in rotating apolar systems persists even at arbitrarily low noise.

Supplementary Note 6. Effects of concentration fluctuations

In this section we will study the effects of concentration fluctuations in a chiral phase. Specifically, we will assess whether in these phases the concentration fluctuations are scale as in all equilibrium systems or are larger as in many active orientationally ordered systems i.e. whether R.M.S. fluctuations of the number of particles $\sqrt{(\delta N)^2}$ in a region containing on average N particles scales as $\sqrt{N}$ as in equilibrium systems or with a higher power as in many active ones. Schematically, the coupled equations for the angle fluctuations $\delta\theta$ and the conserved concentration fluctuations δc are given by

$$\partial_t \theta_q = g(\delta\theta_q, \delta c_q) + \xi_1 \tag{41}$$

$$\partial_t \delta c_q = i\mathbf{q} \cdot \mathbf{J}(\delta\theta_q, \delta c_q)_q + \xi_2 \tag{42}$$

in Fourier space, where the forms of the concentration current $\mathbf{J}_q$ and the function $g(\delta\theta_q, \delta c_q)$ are model-dependent and will be specified in the subsequent subsections and the zero-mean Gaussian white noises ξ_1 and ξ_2 have correlations

$$\langle \xi_1(q,t)\xi_1(q',t') \rangle = 4\pi T_1 \delta(q+q')\delta(t-t') \tag{43}$$

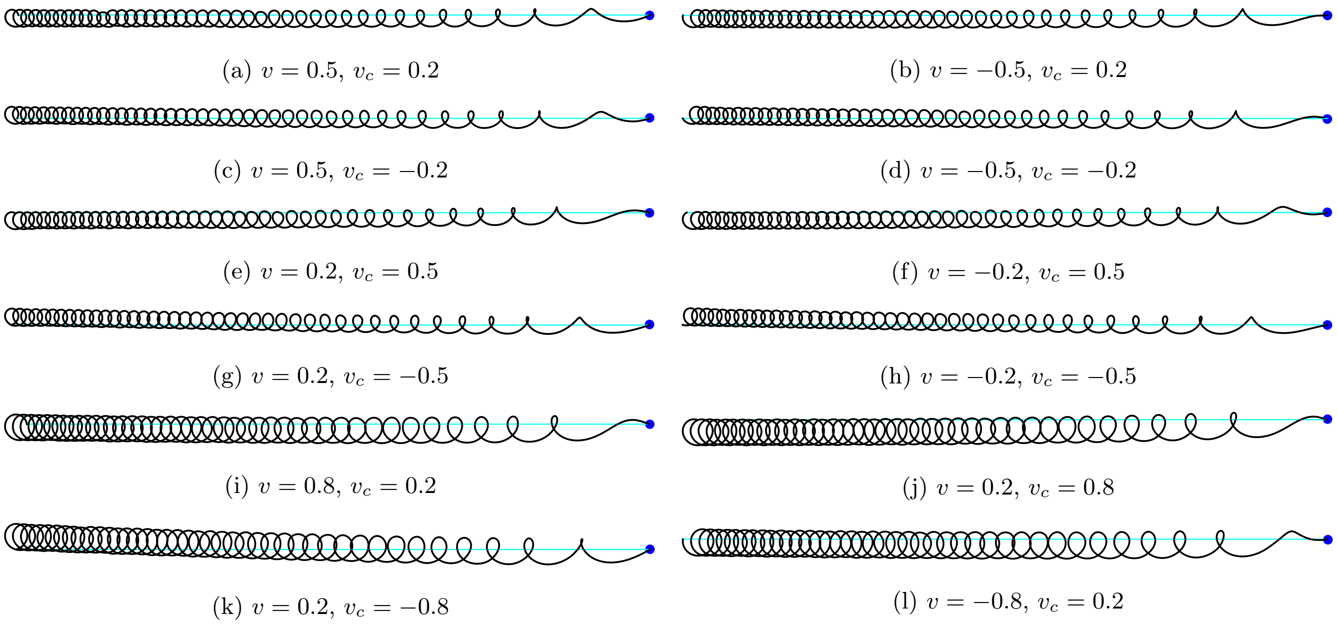

Supplementary Figure 2: Trajectories of recombining $+1/2$ defects for various values of parameters in the units in which $\mu K = 1$, $\Omega = 1$. The cyan line joins the position of the $-1/2$ defect, denoted by the blue dot with the initial position of the $+1/2$ defect.

$$\langle \xi_2(q,t)\xi_2(q',t')\rangle = 4\pi T_2 q^2 \delta(q+q')\delta(t-t'). \tag{44}$$

To deal with these coupled equations in the rotating phase, where $\mathbf{J}_q$ and g are periodic functions of time, we first discuss a consistent scheme for stroboscopic averaging of the time-periodic coefficients in linear equations in Supplementary Note 6 a. We then use this procedure to calculate $\langle \delta c_q(t)\delta c_{-q}(t)\rangle$ which in the limit $q \to 0$ scales as $\delta N^2/N$. In equilibrium, this should be a $\mathcal{O}(1)$ quantity and any dependence of this on N signifies anomalous number fluctuations. We perform this calculation for the six cases discussed earlier in Supplementary Note 6 b and demonstrate that we obtain giant number fluctuations, with $\delta N^2/N \sim N$, only for apolar particles on a substrate in the spinning phase.

a. A perturbative method for high rotation frequency or low activity and concentration fluctuations

In this section we first discuss a general perturbative scheme for stroboscopic averaging of linear dynamical equations with periodic coefficients in Supplementary Note 6 a i and then use this to calculate correlation functions for stochastic linear dynamical systems in Supplementary Note 6 a ii.

i. *Perturbative calculation*

Take a general linear dynamical system

$$\dot{\mathbf{x}} = \mathbf{H}(t)\cdot\mathbf{x} \tag{45}$$

where $\mathbf{H}(t)$ is a periodic matrix, with a period $2\Pi/\Omega$. Ultimately, the vector $\mathbf{x}$ for us will be $(\delta\theta_q, \delta c_q)$. We assume for this averaging that Ω is much larger than all the other inverse time-scales (such as ζ/η) in the problem. Rescaling time $\tau = \Omega t$, we obtain

$$\partial_\tau \mathbf{x} = \varepsilon\tilde{\mathbf{H}}(\tau)\cdot\mathbf{x} \tag{46}$$

where $\epsilon = \zeta/\eta\Omega$ and $\tilde{\mathbf{H}} = \eta\mathbf{H}/\zeta$. We will now review a perturbation scheme that guarantees $\mathcal{O}(\varepsilon^2)$ accuracy up to $\tau \sim \mathcal{O}(\varepsilon)$ or $t \sim \mathcal{O}(\eta/\zeta)$ which is the large timescale in this problem – in the absence of spontaneous rotation, this would be the time scale of active instability. We first recall that by Floquet theorem, the solution of an initial value problem given by (46) can be written as

$$\mathbf{x}(t) = \mathbf{P}(\tau,\varepsilon)e^{\mathbf{B}(\varepsilon)\tau}\mathbf{x}(0) \tag{47}$$

where $\mathbf{P}$ is a 2×2 matrix which is 2π periodic in τ and $\mathbf{B}$ is a τ-independent matrix depending on ε whose eigenvalues are the Floquet exponents. The long-time dynamics and the stability of the system is governed by $\mathbf{B}$.

To perform the stroboscopic average and obtain a time-independent linear equation, we now perform a linear transformation [10]

$$\mathbf{x} = \mathbf{G}\cdot\mathbf{y} = [\mathbf{I} + \mathbf{W}(\tau)]\cdot\mathbf{y} \tag{48}$$

where $\mathbf{I}$ is the identity matrix and $\mathbf{W}(\tau)$ is defined as

$$\mathbf{W}(\tau) = \int_0^\tau [\tilde{\mathbf{H}}(s) - \tilde{\mathbf{H}}^0]ds \tag{49}$$

with

$$\tilde{\mathbf{H}}^0 = \frac{1}{2\pi}\int_0^{2\pi}\tilde{\mathbf{H}}(s)ds. \tag{50}$$

Then

$$\dot{\mathbf{y}} = \mathbf{G}^{-1}\cdot[\tilde{\mathbf{H}}\cdot\mathbf{G} - \partial_\tau\mathbf{G}]\cdot\mathbf{y} = [\varepsilon\tilde{\mathbf{H}}^0 + \varepsilon^2\tilde{\mathbf{H}}^1 + \mathcal{O}(\varepsilon^3)]\cdot\mathbf{y} = \mathbf{L}\cdot\mathbf{y} \tag{51}$$

where

$$\tilde{\mathbf{H}}^1 = \frac{1}{2\pi}\int_0^{2\pi}[\tilde{\mathbf{H}}(\tau)\cdot\mathbf{W}(\tau) - \mathbf{W}(\tau)\cdot\tilde{\mathbf{H}}^0]d\tau. \tag{52}$$

Note that $\tilde{\mathbf{H}}^0$, $\tilde{\mathbf{H}}^1$ and $\mathbf{L}$ are time-independent matrices and the new differential equation in terms of $\mathbf{y}$ has time-independent coefficients and can be trivially solved.

ii. Concentration fluctuations

We now add noise to (45) as

$$\dot{\mathbf{x}} = \mathbf{H}\cdot\mathbf{x} + \boldsymbol{\xi}. \tag{53}$$

As discussed in the introduction of this section, $\mathbf{x} = (\delta\theta_q, \delta c_q)$ and $\boldsymbol{\xi} = (\xi_1, \xi_2)$ whose correlations are given by (43) and (44). While going from $\mathbf{x}$ to $\mathbf{y}$, the noise vector has to be multiplied by $[\mathbf{I} - \varepsilon\mathbf{W}(\tau)]$.

We would like to calculate the concentration correlation function. For this we have to transform back to the variable $\mathbf{x}$ and the original time variable t. However, the correlation functions between $\mathbf{x}$ variables and $\mathbf{y}$ variables are related as

$$\langle\mathbf{y}(t)\mathbf{y}(t)\rangle + 2\varepsilon\langle\mathbf{W}(t)\cdot\mathbf{y}(t)\mathbf{y}(t)\rangle + \mathcal{O}(\varepsilon^2) = \langle\mathbf{x}(t)\mathbf{x}(t)\rangle \tag{54}$$

We are interested in the scaling of the concentration correlation function at small q and the $\mathcal{O}(\varepsilon)$ term in (54) can not modify this $q \to 0$ scaling since $\mathbf{W}(t)$ is at least $\mathcal{O}(q^0)$ and therefore, $\langle\mathbf{W}(t)\cdot\mathbf{y}(t)\mathbf{y}(t)\rangle$ can at most be of the same order in q as $\langle\mathbf{y}(t)\mathbf{y}(t)\rangle$ as $q \to 0$. An argument paralleling this shows that the $\varepsilon\mathbf{W}(t)$ term multiplying the noise can not modify the scaling of the correlation function to leading order in q either. Thus, the correlation function $\langle\mathbf{y}(t)\mathbf{y}(t)\rangle$ where the average denotes average over the realisations of the original noise $\boldsymbol{\xi}$, will have the same leading order in q behaviour (with different prefactors however) as $\langle\mathbf{x}(t)\mathbf{x}(t)\rangle$

This implies that the concentration fluctuations will scale as

$$\langle \delta c_q(t)\delta c_{-q}(t)\rangle \sim T_2 q^2 \left[\frac{\mathrm{Re}[\lambda_1+\lambda_2]|\mathsf{L}_{22}|^2 + (|\lambda_2|^2\mathrm{Re}[\lambda_1] + |\lambda_1|^2\mathrm{Re}[\lambda_2])}{(\lambda_2+\lambda_1^*)(\lambda_1+\lambda_2^*)\mathrm{Re}[\lambda_1]\mathrm{Re}[\lambda_2]}\right] + T_1\left[\frac{\mathrm{Re}[\lambda_1+\lambda_2]|\mathsf{L}_{21}|^2}{(\lambda_2+\lambda_1^*)(\lambda_1+\lambda_2^*)\mathrm{Re}[\lambda_1]\mathrm{Re}[\lambda_2]}\right] \tag{55}$$

where $-\lambda_1$ and $-\lambda_2$ are the eigenvalues of L, the star denotes complex conjugation and Re denotes the real part of a complex quantity. The first term of this expression, $\propto T_2$ is due to the conserving noise in the concentration equation and due to the conserving nature of the concentration fluctuations can be shown to always scale as $\sim q^0$. Any anomalous fluctuation has to be due to the second part of the expression, $\propto T_1$, which is due to the non-conserving noise in the angle equation, scaling as some negative power of q for $q \to 0$. Therefore, we will just have to calculate the eigenvalues of L and L_{21} to check whether our system has anomalous number fluctuations.

b. Equations of motion including the effects of concentration fluctuations

In this section, we will in turn consider apolar and polar suspensions in two-dimensional free-standing films, and in films exchanging momentum with either a three-dimensional fluid or a substrate. We will show that only apolar suspensions on substrates display giant number fluctuations.

i. Two dimensional free-standing film: apolar

As earlier, we will use this system to set-up the apolar calculation and then discuss how other dissipation mechanisms modify this picture in the subsequent sections. The free-energy including terms coupling the order-parameter and the concentration is

$$\mathcal{H} = \int d^2\mathbf{r}[(\alpha/2)\mathbf{Q}:\mathbf{Q} + (\beta/4)[\mathbf{Q}:\mathbf{Q}]^2 + (K/2)(\nabla\mathbf{Q})^2 + \gamma\mathbf{Q}:\nabla\nabla c + f(c) - \gamma_c(\boldsymbol{\epsilon}\cdot\mathbf{Q}):\nabla\nabla c] \tag{56}$$

where $f(c)$ is a function purely of the concentration.

The dynamics of the apolar order parameter is still described by (2), and the force balance equation is only from (3) in Supplementary Note 2 a in that the active stresses can also be functions of concentration, and now read

$$-\eta\nabla^2\mathbf{v} = -\nabla\Pi + \zeta\nabla\cdot(c\mathbf{Q}) - \zeta_c\nabla\cdot(c\boldsymbol{\epsilon}\cdot\mathbf{Q}), \tag{57}$$

We now construct the continuity equation for the concentration $\partial_t c = \nabla\cdot\mathbf{J}$. Based on symmetry arguments,

$$\mathbf{J} = \nabla\frac{\delta\mathcal{H}}{\delta c} + w\nabla\cdot\mathbf{Q} - w_2\boldsymbol{\epsilon}\cdot\nabla\cdot\mathbf{Q} - w_3(\boldsymbol{\epsilon}\cdot\mathbf{Q})\cdot(\nabla\cdot\mathbf{Q}), \tag{58}$$

where we have rescaled the kinetic coefficient relating the chemical potential to the current to 1. Eq. (58) contains active couplings to the orientation, with the coefficients w, w_2 and w_3 in addition to the gradient of the chemical potential [11]. While the current with the coefficient w is allowed in achiral systems, the other two are only possible in chiral ones. The physics of the new mass-currents can be understood as follows: $\nabla\cdot\mathbf{Q}$ implies a local polarity and any active system will have a current along (or opposite to, depending on the sign of w) this direction. In addition a two-dimensional chiral system breaks the left-right symmetry with respect to a given direction therefore implying that a local polar distortion can drift sideways. A fourth active (but achiral) contribution, proportional to $\mathbf{Q}\cdot\nabla\cdot\mathbf{Q}$ was shown to lead to a divergence-free current for small fluctuations about an ordered phase [13]. We now display the linearised dynamical equation for small perturbations about a state with with a constant order-parameter magnitude $S = S_0$ and concentration $c = c_0$. As usual, we write $\theta(\mathbf{r},t) = \Omega t + \delta\theta(\mathbf{r},t)$, and solving for the velocity in terms of the angle and concentration fields, we find the coupled equations

$$\partial_t\begin{pmatrix}\delta\theta\\ \delta c\end{pmatrix} = \begin{pmatrix}M_{\theta\theta} & M_{\theta c}\\ M_{c\theta} & M_{cc}\end{pmatrix}\begin{pmatrix}\delta\theta\\ \delta c\end{pmatrix} \tag{59}$$

where

$$M_{\theta\theta} = -\frac{c_0}{2\eta}[\zeta\cos 2(\phi-\Omega t)+\zeta_c\sin 2(\phi-\Omega t)][1+\lambda\cos 2(\phi-\Omega t)-\lambda_c\sin 2(\phi-\Omega t)]-\Gamma_Q K q^2 \tag{60a}$$

$$M_{\theta c} = -\frac{1}{4\eta}[\zeta_c\cos 2(\phi-\Omega t)-\zeta\sin 2(\phi-\Omega t)][1+\lambda\cos 2(\phi-\Omega t)-\lambda_c\sin 2(\phi-\Omega t)]+\frac{\Gamma_Q}{2}q^2[\gamma\sin 2(\phi-\Omega t)+\gamma_c\cos 2(\phi-\Omega t)] \tag{60b}$$

$$M_{c\theta} = -\frac{q^2}{2}[w_3-2w_2\cos 2(\phi-\Omega t)+2w\sin 2(\phi-\Omega t)] \tag{60c}$$

$$M_{cc} = -D_c q^2 \tag{60d}$$

with D_c being the concentration diffusivity appearing from the term $\nabla^2\delta\mathcal{H}/\delta c$ in the concentration equation.

To lowest order in the perturbation theory in Supplementary Note 6 a, we obtain

$$\mathbf{L} = \begin{pmatrix} -\frac{c_0(\zeta\lambda-\zeta_c\lambda_c)}{4\eta} & -\frac{\zeta_c\lambda+\zeta\lambda_c}{8\eta} \\ -\frac{q^2 w_3}{2} & -D_c q^2 \end{pmatrix} \tag{61}$$

The first eigenvalue of this, λ_1 scales as $\mathcal{O}(q^0)$ for small q:

$$\lambda_1 = -\frac{c_0(\zeta\lambda-\zeta_c\lambda_c)}{4\eta} \tag{62}$$

while the second eigenvalue scales as $\mathcal{O}(q^2)$ for small q:

$$\lambda_2 = -D_c q^2 + \frac{w_3(\zeta_c\lambda+\zeta\lambda_c)}{4c_0(\zeta\lambda-\zeta_c\lambda_c)}q^2 \tag{63}$$

Assuming the second term is small compared to the first one, the system is stable. However, the concentration in the stable phase is seen to be normal by simple power counting and using (55) – in fact, the second part of that expression now scales as $\mathcal{O}(q^2)$, which is subdominant to the first part. If we proceed to the next order in perturbation theory, neither the scaling of the eigenvalues nor the scaling of $\mathbf{L}_{21}$ changes, though their forms do – $\mathbf{L}_{21}$ now also depends on w_1 and w_2 for instance. However, since the scaling with q does not change neither does the scaling of the static structure factor of concentration fluctuations. This implies that this ordered rotating phase has only normal number fluctuations.

ii. *Two-dimensional monolayer at the interface between three-dimensional films: apolar*

The form of the active stresses are again only modified relative to Supplementary Note 2 b in that they can also depend on the concentration c and the equation of the concentration current is the same as the one in Supplementary Note 6 b i. The coupled equations for concentration and angular fluctuations are

$$\partial_t \begin{pmatrix}\delta\theta\\ \delta c\end{pmatrix} = \begin{pmatrix} M_{\theta\theta} & M_{\theta c} \\ M_{c\theta} & M_{cc}\end{pmatrix}\begin{pmatrix}\delta\theta\\ \delta c\end{pmatrix} \tag{64}$$

$$M_{\theta\theta} = -\frac{c_0|q|}{8\eta}[\zeta\cos 2(\phi-\Omega t)+\zeta_c\sin 2(\phi-\Omega t)][1+\lambda\cos 2(\phi-\Omega t)-\lambda_c\sin 2(\phi-\Omega t)]-\Gamma_Q K q^2 \tag{65a}$$

$$M_{\theta c} = -\frac{|q|}{16\eta}[\zeta_c\cos 2(\phi-\Omega t)-\zeta\sin 2(\phi-\Omega t)][1+\lambda\cos 2(\phi-\Omega t)-\lambda_c\sin 2(\phi-\Omega t)]+\frac{\Gamma_Q}{2}q^2[\gamma\sin 2(\phi-\Omega t)+\gamma_c\cos 2(\phi-\Omega t)] \tag{65b}$$

$$M_{c\theta} = -\frac{q^2}{2}[w_3-2w_2\cos 2(\phi-\Omega t)+2w\sin 2(\phi-\Omega t)] \tag{65c}$$

$$M_{cc} = -D_c q^2 \tag{65d}$$

To lowest order in the perturbation theory Supplementary Note 6 a,

$$\mathsf{L} = \begin{pmatrix} -\frac{c_0|q|(\zeta\lambda-\zeta_c\lambda_c)}{16\eta} & -\frac{|q|(\zeta_c\lambda+\zeta\lambda_c)}{32\eta} \\ -\frac{q^2 w_3}{2} & -D_c q^2 \end{pmatrix} \tag{66}$$

The first eigenvalue of this, λ_1 scales as $\mathcal{O}(q)$ for small q:

$$\lambda_1 = -|q|\frac{c_0(\zeta\lambda - \zeta_c\lambda_c)}{16\eta} \tag{67}$$

while the second eigenvalue scales as $\mathcal{O}(q^2)$ for small q:

$$\lambda_2 = -D_c q^2 + \frac{w_3(\zeta_c\lambda + \zeta\lambda_c)}{4c_0(\zeta\lambda - \zeta_c\lambda_c)} q^2 \tag{68}$$

Simple power counting and the use of (55) shows that the number fluctuations are normal with the second part of that expression now scaling as $\mathcal{O}(q^0)$. Going to the next order in perturbation theory does not change the conclusion about the scaling of the number fluctuations.

iii. Two-dimensional monolayer on a substrate: apolar

In this case, in addition to the extra angular fluctuation dependent active force in Supplementary Note 2 c we also have extra concentration-dependent active forces. The force balance equation reads

$$\Gamma\mathbf{v} = -\nabla\Pi + \zeta\nabla\cdot\mathbf{Q} - \zeta_c\nabla\cdot\boldsymbol{\epsilon}\cdot\mathbf{Q} - 2\zeta_2\mathbf{Q}\cdot(\nabla\cdot\mathbf{Q} + \zeta_3\mathbf{Q}\cdot\nabla c + \frac{\zeta_4}{2}\epsilon\cdot\nabla c + \zeta_5(\epsilon\cdot\mathbf{Q})\cdot\nabla c, \tag{69}$$

Eliminating velocity, we obtain the coupled equations for $\delta\theta$ and δc in a perfectly aligned phase with uniform concentration c_0:

$$\partial_t\begin{pmatrix}\delta\theta\\ \delta c\end{pmatrix} = -q^2\begin{pmatrix} M_{\theta\theta} & M_{\theta c} \\ M_{c\theta} & M_{cc}\end{pmatrix}\begin{pmatrix}\delta\theta\\ \delta c\end{pmatrix} \tag{70}$$

$$M_{\theta\theta} = \frac{1}{2\Gamma}[\zeta_2 + \zeta\cos 2(\phi-\Omega t) + \zeta_c\sin 2(\phi-\Omega t)][1+\lambda\cos 2(\phi-\Omega t) - \lambda_c\sin 2(\phi-\Omega t)] - \Gamma_Q K q^2 \tag{71a}$$

$$\begin{aligned} M_{\theta c} = &\frac{\Gamma_Q}{2}[\gamma\sin 2(\phi-\Omega t) + \gamma_c\cos(2(\phi-\Omega t)] \\ &- \frac{1}{4\Gamma}[\zeta_4 + \zeta_5\cos 2(\phi-\Omega t) + \zeta_3\sin 2(\phi-\Omega t)][1+\lambda\cos 2(\phi-\Omega t) - \lambda_c\sin 2(\phi-\Omega t)]\end{aligned} \tag{71b}$$

$$M_{c\theta} = \frac{1}{2}[w_3 - 2w_2\cos 2(\phi-\Omega t) + 2w\sin 2(\phi-\Omega t)] \tag{71c}$$

$$M_{cc} = D_c \tag{71d}$$

To the lowest order in the perturbation theory Supplementary Note 6 a, the averaged coefficient matrix is given by

$$\mathsf{L} = -q^2\begin{pmatrix} \frac{2\zeta_2+\zeta_1\lambda-\zeta_c\lambda_c}{4\Gamma} + \Gamma_Q K & \frac{\zeta_3\lambda_c - 2\zeta_4 - \zeta_5\lambda}{8\Gamma} \\ \frac{w_3}{2} & D_c\end{pmatrix} \tag{72}$$

Both eigenvalues are $\mathcal{O}(q^2)$ in this case while $\mathsf{L}_{21} \sim q^2$. The explicit expressions are rather complicated and we do not display them here. However, using (55), we immediately see that this implies that the concentration correlation function scales as $\mathcal{O}(q^{-2})$. This scaling remains unmodified even at the next order in the perturbation theory. The anomalous $1/q^2$ scaling of the concentration fluctuations implies that in a region containing on average N particles, the standard deviation in the number ΔN scales linearly with N in two dimensions. This is the only scenario that we have examined in which chiral spinning particles display giant number fluctuations. Of course, the orientationally ordered state is not generically unstable in this system in the $\Omega = 0$ limit and the non-rotating ordered state also displays giant number fluctuations. In fact, going beyond the linear theory, the number fluctuations in this case

turns out to be *larger* than in achiral active nematics. In achiral active nematics, the only active concentration current is $\propto \nabla\cdot\mathbf{Q} = \cos 2\theta(\partial_y\theta\hat{x} + \partial_x\theta\hat{y})$. Since active nematics on substrates are only quasi-long range ordered, all such anisotropic terms must average to 0 at large enough system-sizes, i.e., $\langle\cos 2\theta\rangle$ decays as a power of systems size with a typically small exponent η_S. This was shown to lead to a mitigation of the giant number fluctuation with $\Delta N \sim N^{1-\eta_S/2}$ [12]. In chiral apolar systems however, extra concentration currents are allowed, including $(\boldsymbol{\epsilon}\cdot\mathbf{Q})\cdot(\nabla\cdot\mathbf{Q})$ which, when written in terms of θ, is not associated with any anisotropic factor [13]. This implies that this concentration current term does not decrease with increasing system sizes, and consequently, the linear result for number fluctuations is not modified even when nonlinearities are included – i.e. the result $\Delta N \sim N$ is exact in this case.

iv. Two-dimensional momentum conserved systems: polar

We now turn to the examination of polar ordered phases instead of apolar ones. The free-energy now has to be modified from that of Supplementary Note 3 a to include couplings between concentration and polarisation and reads

$$\mathcal{F} = \int d^2\mathbf{r}\left[(\alpha_p/2)p^2 + (\beta_p/4)p^4 + (K_p/2)(\nabla\mathbf{p})^2 + \gamma_p\mathbf{p}\cdot\nabla c - \gamma_{cp}(\boldsymbol{\epsilon}\cdot\mathbf{p})\cdot\nabla c\right]. \tag{73}$$

The terms with the coefficients γ and γ_c that appear in (56) are allowed even in a polar systems but are subdominant to the explicitly polar terms terms with the coefficients γ_p and γ_{cp}. The polarisation equation remains unmodified from the one in Supplementary Note 3 a and the force balance equation is modified only to the extent that the active stresses are also functions of concentration. Finally, the concentration equation is

$$\partial_t c = \nabla\cdot\left[\nabla\frac{\delta\mathcal{F}}{\delta c} + v_p c\mathbf{p} - v_{pc}c\boldsymbol{\epsilon}\cdot\mathbf{p}\right]. \tag{74}$$

This contains two currents $\propto \mathbf{p}$ and $\propto \boldsymbol{\epsilon}\cdot\mathbf{p}$ not present in apolar systems. The former is present in all polar active systems while the latter is only allowed in chiral ones. We have ignored the concentration currents $\propto \nabla\cdot\mathbf{pp}$ since they are subdominant to the explicitly polar currents. We now find the coupled equations for fluctuations about a steadily rotating state with a constant magnitude of polarisation $p_0 = 1$ and uniform concentration c_0:

$$\partial_t\begin{pmatrix}\delta\theta\\ \delta c\end{pmatrix} = \begin{pmatrix}M_{\theta\theta} & M_{\theta c}\\ M_{c\theta} & M_{cc}\end{pmatrix}\begin{pmatrix}\delta\theta\\ \delta c\end{pmatrix}, \tag{75}$$

$$\begin{aligned} M_{\theta\theta} &= -\frac{c_0}{2\eta}[\zeta\cos 2(\phi-\Omega t) + \zeta_c\sin 2(\phi-\Omega t)][1+\lambda\cos 2(\phi-\Omega t) - \lambda_c\sin 2(\phi-\Omega t)] \\ &\quad - [\lambda_1\cos(\phi-\Omega t) - \lambda_2\sin(\phi-\Omega t)]iq - \Gamma_p K_p q^2 && \text{(76a)}\\ M_{\theta c} &= -\frac{1}{4\eta}[\zeta_c\cos 2(\phi-\Omega t) - \zeta\sin 2(\phi-\Omega t)][1+\lambda\cos 2(\phi-\Omega t) - \lambda_c\sin 2(\phi-\Omega t)] \\ &\quad - \Gamma_p[\gamma_{cp}\cos(\phi-\Omega t) + \gamma_p\sin(\phi-\Omega t)]iq && \text{(76b)}\\ M_{c\theta} &= -c_0[v_{pc}\cos(\phi-\Omega t) - v_p\sin(\phi-\Omega t)]iq && \text{(76c)}\\ M_{cc} &= [v_p\cos(\phi-\Omega t) + v_{pc}\sin(\phi-\Omega t)]iq - D_c q^2 && \text{(76d)}\end{aligned}$$

To lowest order in perturbation theory Supplementary Note 6 a, the averaged coefficient matrix is

$$\mathbf{L} = \begin{pmatrix} -\frac{c_0(\zeta\lambda-\zeta_c\lambda_c)}{4\eta} & -\frac{\zeta_c\lambda+\zeta\lambda_c}{8\eta}\\ -0 & -D_c q^2\end{pmatrix} \tag{77}$$

We thus see that to the lowest order, the angular fluctuations do not couple to the concentration. Of course, if we had included the next order in gradient terms in the concentration current, as in the apolar case, a term with the coefficient w_3 as in Supplementary Note 6 b i would have led to a coupling between them. However, as we saw in that case, this would only lead to normal number fluctuations. Furthermore, at the next order in perturbation, a purely imaginary $\mathbf{L}_{21} \sim \mathcal{O}(q)$ emerges. However, this is still insufficient to change the scaling of concentration fluctuations which scales as $\mathcal{O}(q^0)$. Therefore, the polar chiral rotating system in a free-standing film has only normal number

fluctuations.

v. *Two-dimensional monolayer at the interface between three-dimensional fluids: polar*

In this case, the coupled equations for concentration and angular fluctuations become

$$\partial_t \begin{pmatrix} \delta\theta \\ \delta c \end{pmatrix} = \begin{pmatrix} M_{\theta\theta} & M_{\theta c} \\ M_{c\theta} & M_{cc} \end{pmatrix} \begin{pmatrix} \delta\theta \\ \delta c \end{pmatrix}, \tag{78}$$

$$\begin{aligned} M_{\theta\theta} &= -\frac{c_0|q|}{8\eta}[\zeta\cos 2(\phi-\Omega t)+\zeta_c\sin 2(\phi-\Omega t)][1+\lambda\cos 2(\phi-\Omega t)-\lambda_c\sin 2(\phi-\Omega t)] \\ &\quad -[\lambda_1\cos(\phi-\Omega t)-\lambda_2\sin(\phi-\Omega t)]iq-\Gamma_p K_p q^2 \end{aligned} \tag{79a}$$

$$M_{\theta c} = -\frac{|q|}{16\eta}[\zeta_c\cos 2(\phi-\Omega t)-\zeta\sin 2(\phi-\Omega t)][1+\lambda\cos 2(\phi-\Omega t)-\lambda_c\sin 2(\phi-\Omega t)]-\Gamma_p[\gamma_{cp}\cos(\phi-\Omega t)+\gamma_p\sin(\phi-\Omega t)]iq \tag{79b}$$

$$M_{c\theta} = -c_0[v_{pc}\cos(\phi-\Omega t)-v_p\sin(\phi-\Omega t)]iq \tag{79c}$$

$$M_{cc} = [v_p\cos(\phi-\Omega t)+v_{pc}\sin(\phi-\Omega t)]iq-D_c q^2 \tag{79d}$$

Using the lowest order approximation in Supplementary Note 6 a, the coefficient matrix is

$$\mathbf{L} = \begin{pmatrix} -\frac{c_0|q|(\zeta\lambda-\zeta_c\lambda_c)}{16\eta} & -\frac{|q|(\zeta_c\lambda+\zeta\lambda_c)}{32\eta} \\ -0 & -D_c q^2 \end{pmatrix} \tag{80}$$

As in Supplementary Note 6 b iv, the coupling of angular fluctuations to concentration field only emerges if we go to the next order in the perturbation theory of Supplementary Note 6 a. However, even at this order, $\lambda_1 \sim q$ and $\lambda_2 \sim q^2$. Furthermore, unlike Supplementary Note 6 b iv, $\mathbf{L}_{21} \sim q^2$ at this order. Using this, in conjunction (55), we see that the equal-time correlator for concentration fluctuations scales as $\mathcal{O}(q^0)$ in this case as well. Therefore, there is no giant number fluctuations in this case.

vi. *Two-dimensional monolayer on a substrate: polar*

The dynamical equation for polarisation is exactly the same as in Supplementary Note 3 c and the concentration equation is identical to the one in Supplementary Note 6 b iv. The coupled equations for angle and concentration fields in this case are

$$\partial_t \begin{pmatrix} \delta\theta \\ \delta c \end{pmatrix} = \begin{pmatrix} M_{\theta\theta} & M_{\theta c} \\ M_{c\theta} & M_{cc} \end{pmatrix} \begin{pmatrix} \delta\theta \\ \delta c \end{pmatrix}, \tag{81}$$

$$M_{\theta\theta} = \left[-\frac{\Lambda\upsilon}{\Gamma}\sin^2(\phi-\Omega_p t)+\frac{\Lambda\upsilon_c-\Lambda_c\upsilon}{2\Gamma}\sin 2(\phi-\Omega_p t)+\frac{\Lambda_c\upsilon_c}{\Gamma}\cos^2(\phi-\Omega_p t)\right] \tag{82a}$$

$$M_{\theta c} = -\Gamma_p[\gamma_{cp}\cos(\phi-\Omega_p t)+\gamma_p\sin(\phi-\Omega_p t)]iq \tag{82b}$$

$$M_{c\theta} = -c_0[v_{pc}\cos(\phi-\Omega_p t)-v_p\sin(\phi-\Omega_p t)]iq \tag{82c}$$

$$M_{cc} = -D_c q^2 \tag{82d}$$

Using the lowest order approximation in Supplementary Note 6 a, the coefficient matrix is completely decoupled:

$$\mathbf{L} = \begin{pmatrix} -\frac{\Lambda\upsilon-\Lambda_c\upsilon_c}{2\Gamma} & 0 \\ 0 & -D_c q^2 \end{pmatrix} \tag{83}$$

Purely imaginary $\mathcal{O}(q)$ terms are generated in both $\mathbf{L}_{12}$ and $\mathbf{L}_{21}$ at the next order. However, this is insufficient to modify the scaling of the equal-time correlator for concentration fluctuations which goes as $\sim q^0$. Therefore, the number fluctuations remain normal even for a chiral polar suspension on a substrate. Despite the similarity of this

with Supplementary Note 6 b iv, the underlying reason for there not being giant number fluctuations in this case is distinct and rely on the active motility and passive coupling of the polarisation to the velocity (and not its gradient) as we explain more fully in [4].

---

* ananyo.maitra@u-psud.fr
† martin.lenz@u-psud.fr

Supplementary References

[1] Simha, R.A., Ramaswamy, S. Hydrodynamic fluctuations and instabilities in ordered suspensions of self-propelled particles. *Phys. Rev. Lett.* **89,** 058101 (2002)
[2] Marchetti, M. C. et al. Hydrodynamics of soft active matter. *Rev. Mod. Phys* **85,** 1143 (2013)
[3] Liebchen, B. & Levis, D. Collective Behavior of Chiral Active Matter: Pattern Formation and Enhanced Flocking. *Phys. Rev. Lett* 119, 058002 (2017)
[4] Maitra A., Srivastava P., Marchetti M. C., Ramaswamy S. & Lenz M. Swimmer suspensions on substrates: anomalous stability and long-range order. arXiv: 1901.01069
[5] Pismen, L. Dynamics of defects in an active nematic layer. *Phys. Rev. E* **88,** 050502 (2013)
[6] Giomi, L., Bowick, M. J., Mishra, P., Sknepnek, R. & Marchetti, M. C. *Philos. Trans. Royal Soc. A* **372,** 20130365 (2014)
[7] Kats, E., Lebedev, V. V & Malinin, S. V. Disclination Motion in Liquid Crystalline Films. *J. Exp. Theor. Phys.* **95,** 714 (2002)
[8] Denniston, C. Disclination dynamics in nematic liquid crystals. *Phys. Rev. B* **54,** 6272 (1996)
[9] Ryskin, G. & Kremenetsky, M. Drag force on a line defect moving through an otherwise undisturbed field: Disclination line in a nematic liquid crystal. *Phys. Rev. Lett.* **67,** 1574 (1991)
[10] Hartono &van der Burgh, A. H. P. Higher-order averaging: periodic solutions, linear systems and an application. *Nonlinear Anal. Theory Methods Appl.* **52,** 1727-1744 (2003)
[11] Ramaswamy, S., Simha, R. A.,Toner, J. Active nematics on a substrate: Giant number fluctuations and long-time tails. *Europhys. Lett* **62,** 196 (2003)
[12] Shankar, S., Ramaswamy, S. & Marchetti, M. C. Low-noise phase of a two-dimensional active nematic system. *Phys. Rev. E* **97,** 012707 (2018)
[13] Maitra A., Srivastava P., Lintuvuori, J., Marchetti M. C., Ramaswamy S. & Lenz M. A nonequilibrium force can stabilize 2D active nematics. *Proc. Natl. Acad. Sci. USA* **115,** 6934-6939 (2018)
[14] Shankar S., Ramaswamy S., Marchetti M.C. & Bowick M. J., Defect Unbinding in Active Nematics. *Phys. Rev. Lett.* **121,** 108002 (2018)
[15] Chajwa, R., Menon, N. & Ramaswamy, S. Kepler orbits of settling discs. arXiv: 1803.10269 (2018)
